# Tumor response assessment by MRI following stereotactic body radiation therapy for hepatocellular carcinoma

**DOI:** 10.1371/journal.pone.0176118

**Published:** 2017-04-25

**Authors:** Guillaume Oldrini, Andres Huertas, Sophie Renard-Oldrini, Hélène Taste-George, Guillaume Vogin, Valérie Laurent, Julia Salleron, Philippe Henrot

**Affiliations:** 1 Service d’Imagerie, Institut de cancérologie de Lorraine, Vandoeuvre-lès-Nancy Cedex, France; 2 Service de radiothérapie, Institut de cancérologie de Lorraine, Vandoeuvre-lès-Nancy Cedex, France; 3 Département de radiologie Adulte, CHU Nancy, Vandoeuvre-lès-Nancy, France; 4 Cellule Data Biostatistique, Institut de cancérologie de Lorraine, Vandoeuvre-lès-Nancy Cedex, France; 5 Service d’Imagerie, Institut de cancérologie de Lorraine, Vandoeuvre-lès-Nancy Cedex, France; University of Michigan, UNITED STATES

## Abstract

**Background:**

To evaluate the MRI features of a tumor response, local control, and predictive factors of local control after stereotactic body radiation therapy (SBRT) for hepatocellular carcinoma (HCC).

**Methods:**

Thirty-five consecutive patients with 48 HCCs who were treated by SBRT were included in this retrospective study. All patients provided written informed consent to be treated by SBRT, and prior to inclusion they authorized use of the treatment data for further studies. The assessment was made using MRI, with determination of local and hepatic responses according to Response Evaluation Criteria in Solid Tumors (RECIST) and modified RECIST (mRECIST) criteria during a two-year follow-up.

**Results:**

The local response rate according to mRECIST was higher than with RECIST. A tumor diameter less than 20 mm at baseline was an independent predictive factor for RECIST and mRECIST responses, as was diffusion-weighted signal for RECIST. During follow-up, a tumor diameter of <20 mm (*p* = 0.034) and absence of a high intensity on T2-weighted (*p* = 0.006) and diffusion-weighted images (*p* = 0.039) were associated with a better response according to RECIST. Post-treatment changes include peritumoral ring-like enhanced changes with high intensity on T2-weighted images.

**Conclusions:**

SBRT is a promising technique for the treatment of inoperable HCC. Post-treatment changes on MRI images can resemble tumor progression and as such must be adequately distinguished. The regression of tumorous enhancement is variable over time, although diffusion-weighted and T2-weighted intensities are predictive factors for tumor RECIST responses on subsequent MRIs. They hence provide a way to reliably predict treatment responses.

## Introduction

Hepatocellular carcinoma (HCC) is the most frequently occurring primary hepatic tumor, developing in association with advanced cirrhosis in 90% of cases. It accounts for approximately 800,000 new cases annually, making it the fifth most common cancer in men and the ninth in women [1, 2].

Surgical resection and orthotopic liver transplantation are the standard of care for solitary liver-confined HCCs. For inoperable tumors, radiofrequency ablation (RFA) and percutaneous ethanol injection (PEI) are the only recommended treatments with curative intent, while transarterial chemoembolization (TACE) is generally seen as a palliative therapy [3].

International guidelines do not indicate that radiotherapy is an efficient and validated treatment for HCC [4], since conventional radiotherapy is typically associated with high rates of liver toxicity and a low efficacy [5–7], although three-dimensional conformal radiotherapy has nonetheless been shown to yield encouraging results [8–10]. Stereotactic body radiation therapy (SBRT) delivers high-dose selective irradiation with millimeter precision to a small volume. The Cyberknife® is a robotic SBRT dedicated device that can deliver 100 to 200 photon beams to the target. Two orthogonal X-ray tubes allow for real-time tracking of the tumor, thus allowing the beams to take into account respiratory movements.

SBRT is responsible for changes in local post-treatment images that have not been described in detail in the literature. The aim of this work was to describe SBRT’s different presentations, to study predictive factors for tumor responses following treatment, and to compare tumor response assessments according to the Response Evaluation Criteria in Solid Tumors (RECIST) v1.1 and modified RECIST (mRECIST) methods. Our study highlights SBRT as a promising technique for the treatment of inoperable HCC and demonstrates that post-treatment changes on MRI images can provide a way to reliably predict treatment responses.

## Materials and methods

### Patients

This study was based on retrospective interpretation of prospectively acquired data from October 2011 to April 2013. Clinical data was also retrospectively collected from electronic medical record system. It was conducted with 35 patients who were treated consecutively by SBRT for HCC between October 2011 and April 2013. The eligibility criteria were the standard criteria for treating HCC patients with SBRT: an Eastern Cooperative Oncology Group (ECOG) score of ≤ 2, inoperable tumors (e.g. the patient being unfit for surgery, or tumor-related contraindications), and a maximum tumor diameter of ≤ 6 cm. The HCC diagnosis could be histological or based on the radiological criteria of the American Association for the Study of Liver Diseases (AASLD) [3]. All cases were presented to a multidisciplinary liver tumors board that included hepatologists, hepatic surgeons, radiation oncologists, and radiologists; and they were treated in the setting of a clinical trial for SBRT treatment (NCT01165346) which evaluates percentage of non-progression 18 months after treatment, according to RECIST criteria. All patients provided written and signed informed consent to be treated by SBRT, and prior to inclusion they authorized the use of the treatment data for further studies. This study was approved by the French Data Protection Authority (“Commission Nationale de l’Informatique et des Libertés”).

Eight patients without follow-up by MR imaging were excluded. Twenty-seven patients were studied.

### Treatment

Seven to ten days before the scheduled CT, 2–4 fiducial markers were implanted percutaneously 2 to 5 cm next to the lesion under sonographic guidance following administration of local anesthesia, thus allowing real-time tracking during treatment [11]. The total delivered dose was 45 Gy on the 80% isodose, in three fractions over 10 days. Gross tumor volume (GTV) was defined as the tumor mass that could be discerned by diagnostic imaging. An isotropic margin of 5 mm was added to account for the microscopic extension (clinical target volume, CTV) and another 3 mm margin was added to compensate for uncertainties regarding the position (previsional target volume, PTV).

### Image analysis

All patients had a pre-treatment MRI. After the treatment, follow-up included MR imaging every three months during the first two years.

Pre and post-treatment imaging were performed on different 1.5 Tesla MRI instruments, depending on the hospital that provided the treatment. These included axial fat-saturated T2-weighted sequences, axial diffusion-weighted sequences (b = 600 s/mm^2^), axial IP/OP sequences, and axial gadolinium-enhanced dynamic multiphase sequences.

All images were reviewed by the same senior radiologist (GO, 5 years experience with cancer imaging) on our picture archiving and communication system (PACS) (Impax, Agfa). The radiologist was blinded to the MRI reports. All MRIs were evaluated in terms of T2-weighted intensity (the reference tissue was normal peritumoral liver parenchyma), diffusion-weighted intensity (the reference tissue was normal peritumoral liver parenchyma), tumor vascular enhancement, tumor size (on arterial phase), and sub-capsular location (defined as a distance less than 1 cm between the lesion and the capsule). Additionally, all post-treatment MRIs were also evaluated in terms of the occurrence of a new lesion, peritumoral changes, their dimensions, and the presence of a capsular retraction. For the post-treatment imaging, tumor response assessment according to RECIST v1.1 and mRECIST was performed, determining infield progression (occurring within the PTV) and outfield progression (intrahepatic recurrence outside the PTV) using dosimetric data. According to the modified RECIST [12], a complete response (CR) was defined as the disappearance of contrast enhancement in the tumor for the arterial phase. A partial response (PR) was defined as at least a 30% volume reduction of an enhanced area in the arterial phase, taking the baseline diameter of the tumor as a reference. Tumors without any of these changes, or an increase in volume, were deemed to represent instances of stable disease, since a prior study by Sanuki et al. [13] found that an increase of at least 20% in the diameter of a viable (enhancing) lesion, using the smallest diameter of the viable lesion as a reference, was considered to represent progressive disease (PD). All data are available on S1 Table.

### Statistical analysis

Quantitative variables were described with median and interquartile ranges; qualitative variables by frequency and percentage.

The modification of each MRI parameter from stereotactic body radiation therapy to the last available MRI was described by the Actuarial method. The incidence of sub-capsular location of the tumor was compared according to the sub-capsular location using the log-rank test.

The incidence of the first complete remission according to RECIST and mRECIST criteria were also described by the Actuarial method. Predictive factors at baseline of complete response were investigated by a Cox proportional hazard model. In order to take into account patients with two tumors, covariance matrices were determined with the robust sandwich assessment method of Lin and Wei [14]. A time-dependent Cox proportional hazard model was performed in order to investigate clinical factors from stereotactic body radiation therapy to the last available MRI. Parameters with a *p*-value of less than 0.2 were introduced into a multivariable Cox model with a significance level for removing effects at 0.05.

All statistical analyses were performed using SAS version 9.3 (SAS, Cary, NC, USA). The significance level was set at 0.05.

## Results

Of the 27 patients who were analyzed, 81.5% (n = 22) were men. The mean patient age was 68 years (SD 11.25 years). Forty-four percent (n = 12) of the patients had a sub-capsular tumor. All patients where cirrhotic, with the most frequent cause of cirrhosis being chronic alcoholism in 41% of cases, while 85% (n = 23) of the patients were Child-Pugh score A. The median initial alpha-fetoprotein (AFP) level was 5 ng/mL [3;21] (Table 1). Median follow-up was 10 months [7;14] and the median number of MRIs per patient was 4 [2;6]. The minimum of follow-up time was 4 months and the maximum was 26 months. Precisely all the 27 patients were followed for more than 3 months, 26 (96.3%) for more than 6 months, 19 (70.4%) for more than 9 months and 12 (44.4%) for more than 12 months. A total of 35 lesions were analyzed: 18 patients (66.7%) exhibited one lesion and 9 patients exhibited two synchronous lesions.

**Table 1:** Characteristics of the patients.

AFP ng/mL (*median and IQR)*	5 [3;21]
Child-Pugh score *%(n)*	
A	85% (23)
B	15% (4)
Etiology of cirrhosis *%(n)*	
Alcoholism	41% (11)
Viral	37% (10)
Metabolic	26% (7)
Hemochromatosis	11% (3)
Number of MRI per patient (*median and IQR)*	4 [3;5]
Number of lesions per patient *%(n)*	
1	66.7%(18)
2	33.3% (9)

Abbreviation: NASH: Nonalcoholic steatohepatitis; IQR: inter-quartile range;% percentage; n frequency; AFP: alpha-fetoprotein

**Fig 1:**
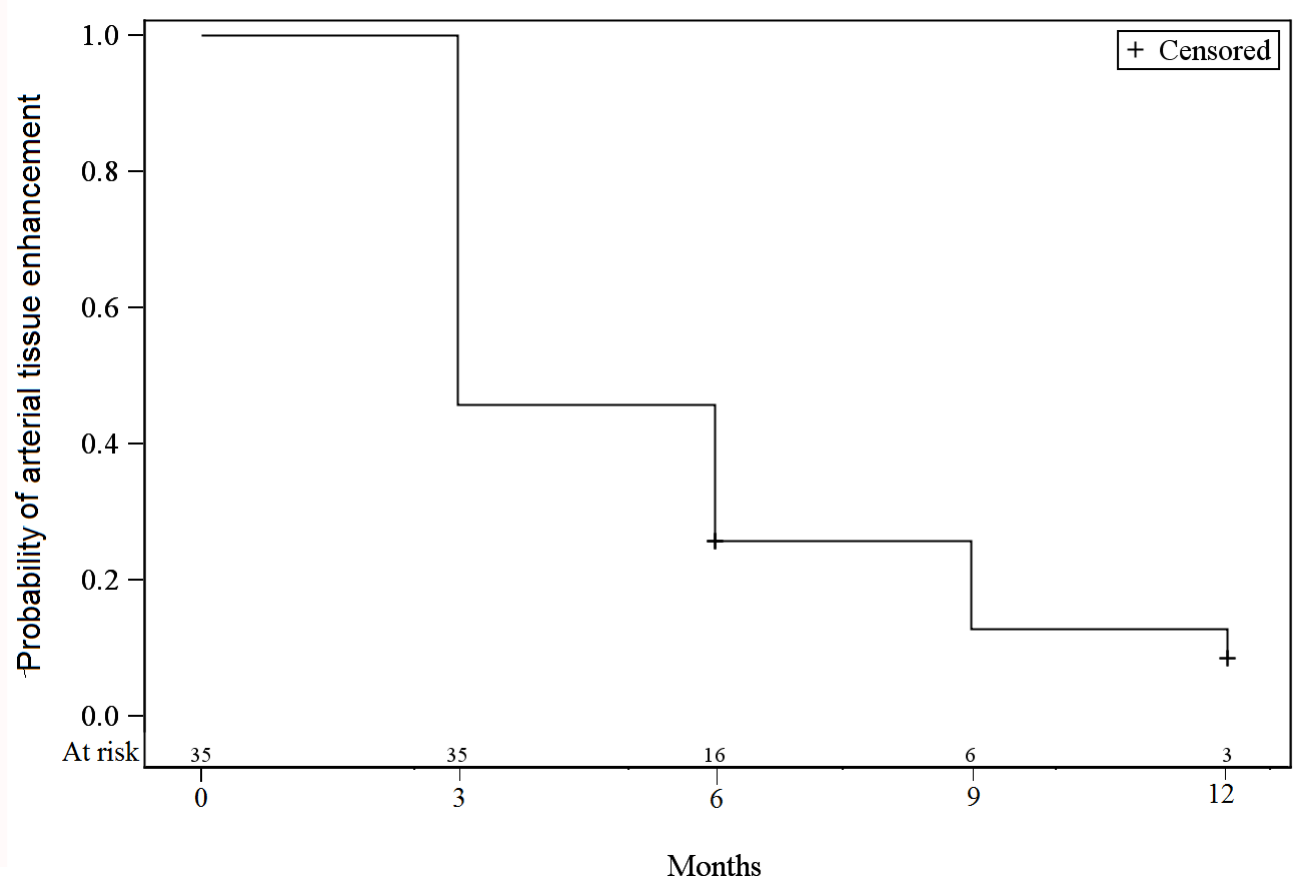
Incidence of tumor enhancement at baseline and during follow-up.

**Fig 2:**
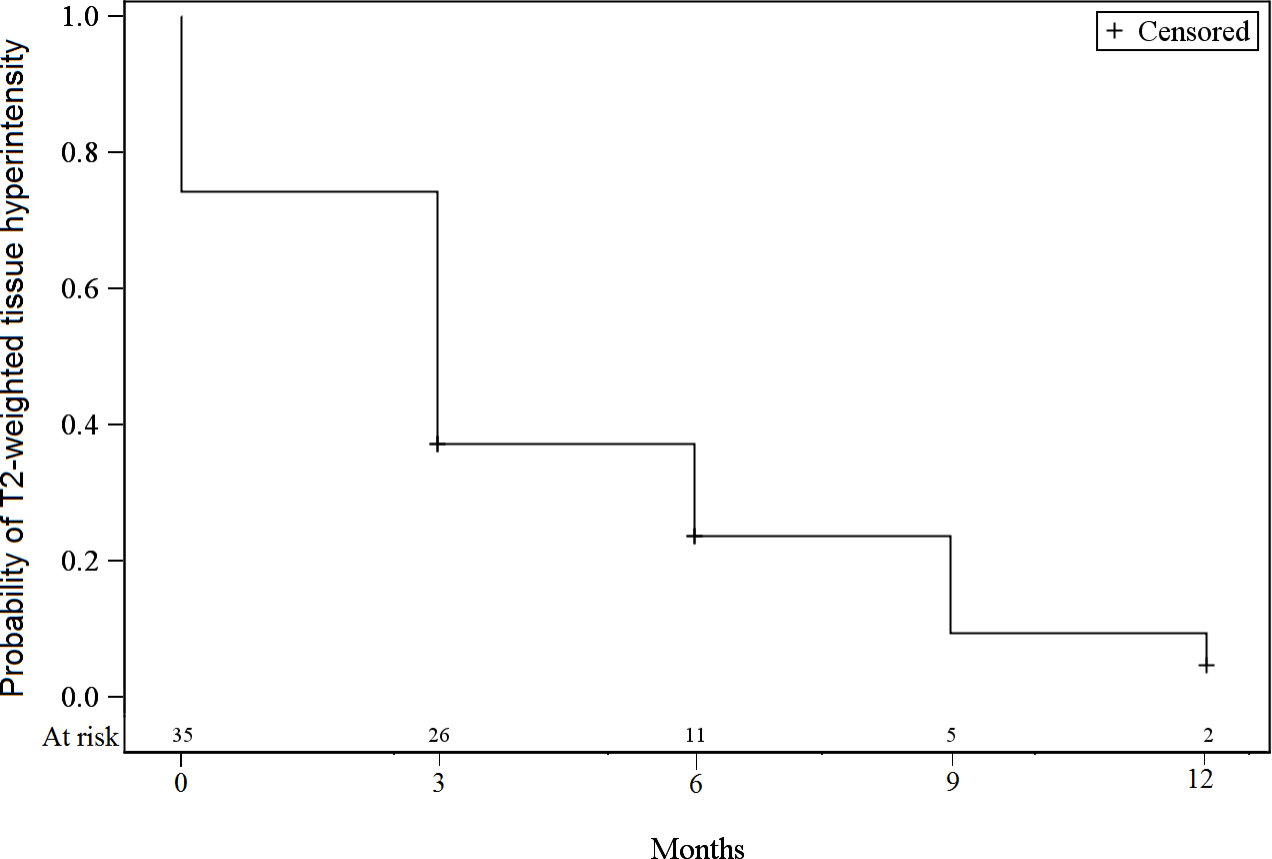
Incidence of T2-weighted hyperintensity at baseline and during follow-up.

**Fig 3:**
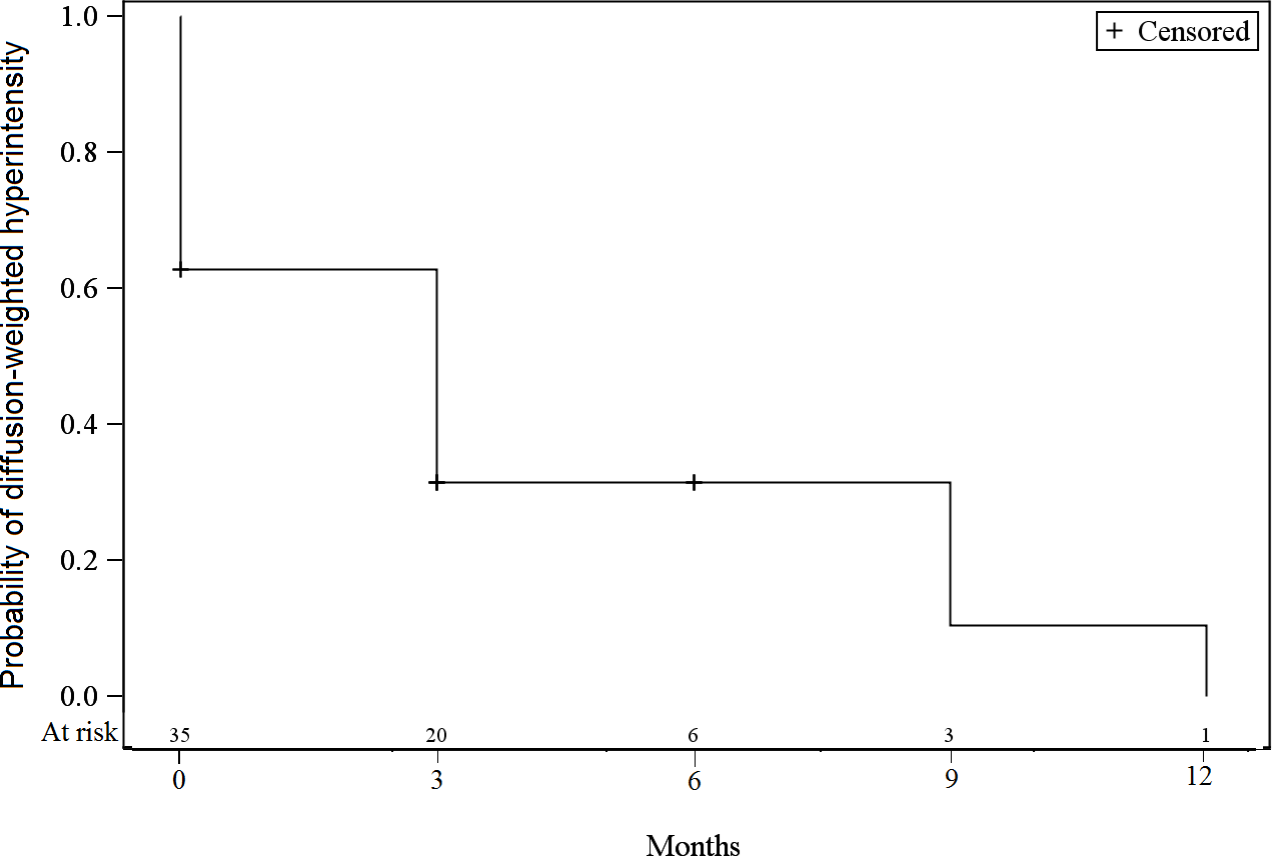
Incidence of diffusion-weighted hyperintensity at baseline and during follow-up.

At baseline, all lesions had an arterial tissue enhancement with a wash-out on portal and/or tardive phase, a T2-weighted tissue hyperintensity was found in 74.3% of the tumors, and a diffusion-weighted hyperintensity was present in 62.9%. The tumorous enhancement disappeared in 54.3% of the lesions at 3 months, and in 74.3% of the lesions at 6 months (Fig 1). The T2-weighted and diffusion-weighted hyperintensities decreased over time (Figs 2 and 3). The median size of the lesions at baseline was 20 mm [12;47]. At 3 months this was 5 mm [0;33], with a progressive decrease of the median size throughout the follow-up period.

**Fig 4:**
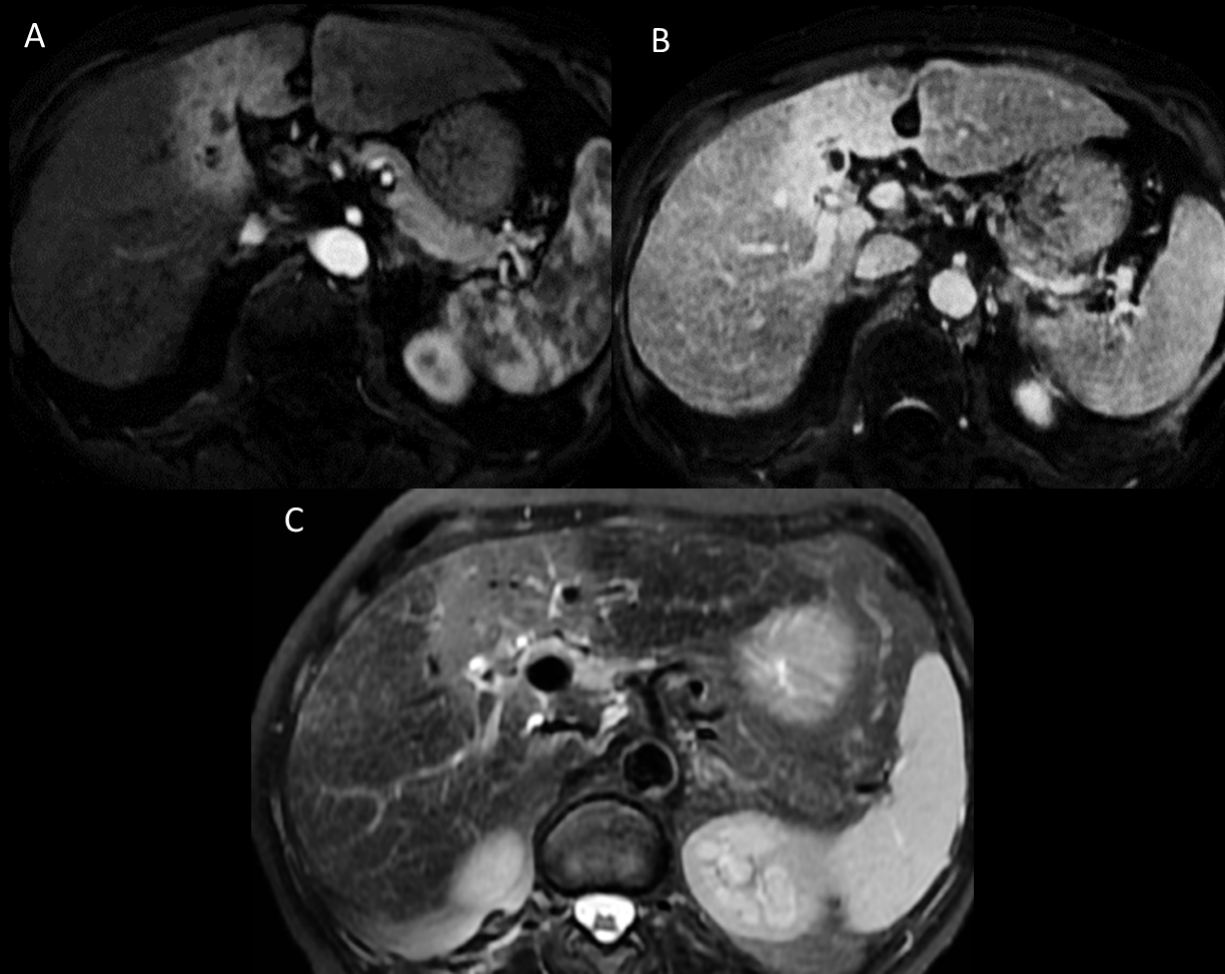
A 48-year-old man with HCC. MRI at three months. 4A Enhanced post-treatment changes on arterial phase. 4B. Enhanced post-treatment changes on venal phase. 4C. T2-weighted hyperintense post-treatment changes.

Perilesional changes following treatment (Fig 4) were discerned as a ring-like enhanced signal on the arterial phase, which persisted on delayed phases. This was also apparent as a T2-weighted hyperintensity, as compared with normal hepatic tissue. These changes regressed steadily. For these 35 lesions, a perilesional ring-like enhanced image was present in 97.1% at 3 months, and in 82.2% of the lesions at 12 months, with a mean diameter decreasing from 60.5 mm to 41.6 mm, respectively, for these two time points. T2-weighted hyperintensity could be seen in 94.2% of the lesions at 3 months, and this decreased to 79.8% at 12 months.

**Fig 5:**
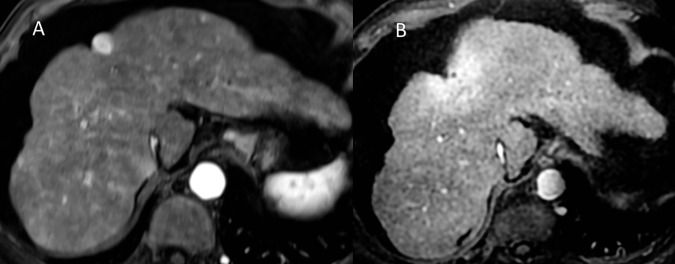
A 52-year-old man with HCC. 5A. Sub-capsular HCC on arterial phase on dynamic 3D T1 weighted imaging at the time of diagnosis. 5B. MRI at six months: portal phase on dynamic 3D T1 weighted imaging. Post-treatment changes with hypervascular ring and capsular retraction.

For the 35 lesions, the cumulative incidence of capsular retraction (Fig 5) was 11.4% at 3 months, 29.8% at 6 months, 49.9% at 9 months, and 57.1% at 12 months. Sub-capsular location of the tumor was significantly predictive of a capsular retraction (Table 2, Log-rank test, *p* = 0.016). Thus, at 6 months, 36.4% of the retraction occurred in the absence of a sub-capsular lesion, versus 63.3% if the lesion was sub-capsular.

**Table 2:** Cox regression analyses of capsular retraction according to sub-capsular location of the tumor.

Sub-capsular lesion	Frequency	Median capsular retraction time (months)	HR and 95% CI	p-value
No	20	12	1	0.016
Yes	15	6	3.33[1.31;8.50]	

Abbreviation: CI, confidence interval; HR, Hazard ratio. P-value was computed with the log-rank test

**Fig 6:**
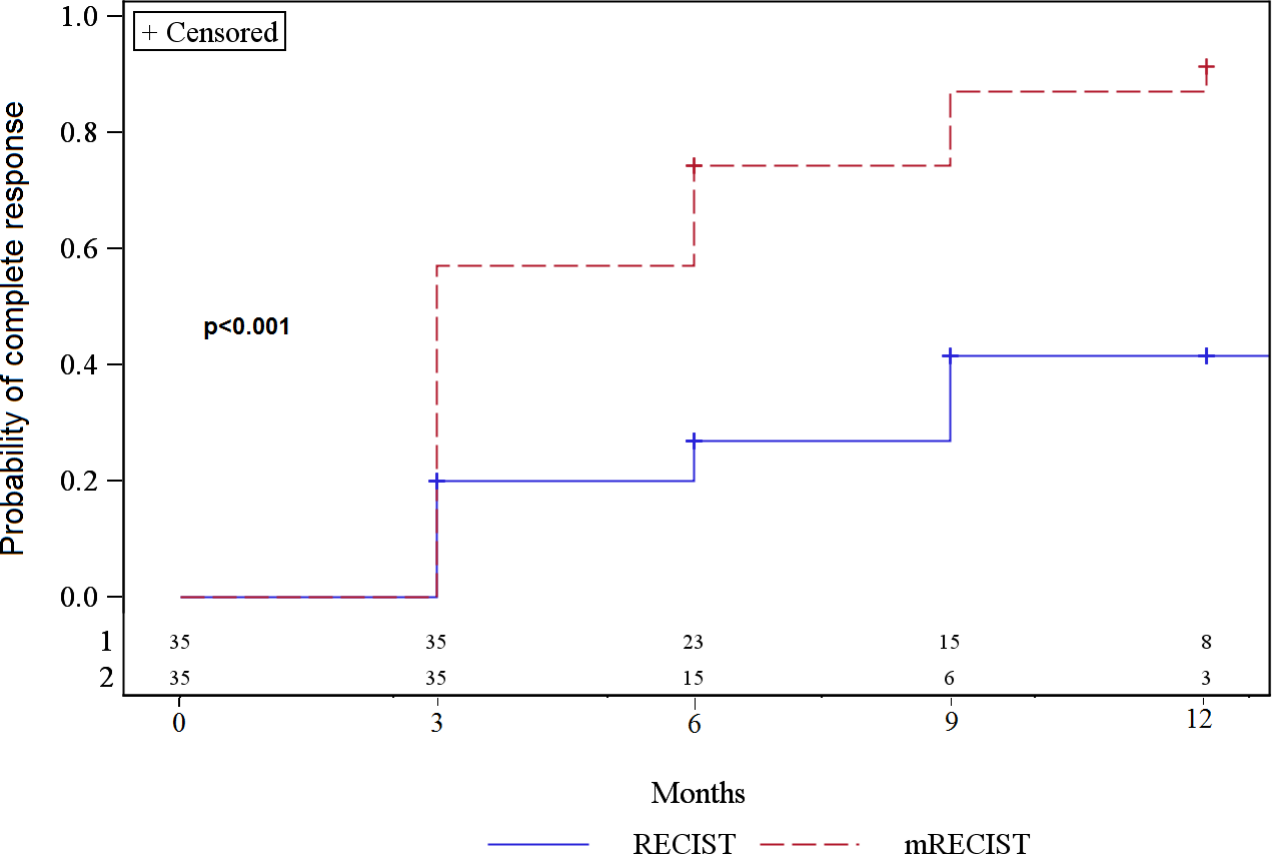
Occurrence of a complete response according to RECIST and mRECIST.

At 3 months, complete response rates according to RECIST and mRECIST were 20% and 57%, respectively; increasing up to 41.6% and 91.4%, respectively, at 12 months (Fig 6). There was a significant difference (Log-rank test, *p*<0.001) in the frequency of complete responses according to the RECIST versus the mRECIST method. Only one infield progression was found (2.8%) during follow-up, while all other lesions were stable or partial responses according to RECIST and mRECIST.

The total number of lesions, gender, subcapsular location of the lesion, cause of the cirrhosis, Child-Pugh score, T2-weighted signal intensity, and AFP level at baseline did not significantly influence the response rate (Table 3). Diffusion-weighted hyperintensity of the lesion at baseline was a predictive factor for a worse response to the treatment according to RECIST criteria. The absence of a high intensity on diffusion-weighted images at baseline positively influenced the complete response rate according to RECIST (*p* = 0.027). A tumor diameter of < 20 mm at baseline was associated with a better response according to RECIST (*p* = 0.028) and mRECIST (*p* = 0.035). During follow-up, a tumor diameter of <20 mm (*p* = 0.034), and an absence of a high intensity on T2-weighted (*p* = 0.006) and diffusion-weighted images (*p* = 0.039) were associated with a better response according to RECIST. Among the 26 tumors with T2-weighted hyperintensity at baseline, 72.7% (16/22) lost this feature at the time of complete response, versus 50% (2/4) when there was not a complete response. Among the 16 tumors with diffusion weighted hyperintensity at baseline, 64.3% (9/14) lost this feature at the time of a complete response, versus 50% (1/2) when there was not a complete response.

**Table 3:** Cox regression analyses of predictive factors at baseline and during follow-up of complete responses according to RECIST and mRECIST.

		RECIST		mRECIST	
		HR and 95% CI	*p-*value	HR and 95% CI	*p-*value
**Parameters at baseline**					
Number of lesions	1	1	0.843	1	0.613
	2	1.13 [0.33;3.85]		0.89 [0.57;1.39]	
Gender	Male	1	0.193	1	0.189
	Female	2.25 [0.66;7.64]		1.43 [0.84;2.43]	
Alcoholic cirrhosis	No	1	0.7772	1	0.263
	Yes	0.85 [0.27;2.67]		1.27 [0.83;1.94]	
Child-Pugh score	A	1	0.680	1	0.734
	B	1.33 [0.35;5.12]		1.09 [0.67;1.75]	
AFP level*	<20 ng/mL	1.25 [0.35;4.39]	0.733	1.40 [0.82;2.40]	0.223
	≥20 ng/mL	1		1	
Sub-capsular location	No	1	0.762	1	0.200
	Yes	1.19 [0.40;3.56]		0.73 [0.46;1.18]	
Tumor diameter	<20 mm	5.40 [1.20;24.36]	**0.028**	1.7 [1.04;2.79]	**0.035**
	≥20 mm	1		1	
T2-weighted hyperintensity	No	2.63 [0.87;7.95]	0.087	1.25 [0.8;1.96]	0.330
	Yes	1		1	
Diffusion-weighted hyperintensity	No	3.96 [1.17;13.43]	**0.027**	1.27 [0.79;2.04]	0.328
	Yes	1		1	
**Parameters during follow-up**					
Tumor diameter*	<20 mm	8.76 [1.18;65.21]	**0.034**	1.54 [0.98;2.41]	0.062
	≥20 mm	1		1	
T2-weighted hyperintensity*	No	5.84 [1.65;20.6]	**0.006**	1.4 [0.86;2.27]	0.178
	Yes	1		1	
Diffusion-weighted hyperintensity*	No	4.37 [1.07;17.81]	**0.039**	1.49 [0.86;2.57]	0.154
	Yes	1		1	
Enhancement	No	1.5 [0.34;6.55]	0.590	-	-
	Yes	1			

Abbreviation: CI, confidence interval; HR, Hazard ratio; AFP, alpha-fetoprotein level. Baseline parameters were recorded at the date of Stereotactic body radiation therapy. Parameters during follow-up were recorded from 3 months after therapy to the last available MRI or completed responses (event in Cox survival analysis).

*At baseline.

Outfield progression was detected for 10 patients (37%), with the occurrence of a new hepatic lesion outside of the radiation field. The incidence of outfield progression was not significantly correlated with any of the studied factors (e.g. total number of lesions, gender, subcapsular location of the lesion, cause of the cirrhosis, Child-Pugh score, T2-weighted or diffusion-weighted signal intensity, or AFP level at baseline).

## Discussion

The role of SBRT is currently undefined in international guidelines for HCC treatment. It is absent from AASLD and European Association for the Study of the Liver–European Organization for Research and Treatment of Cancer (EASL–EORTC) guidelines, while in the National Comprehensive Cancer Network (NCCN) guidelines it is mentioned as an option for inoperable tumors [15]. However, it exhibited good results for local control [13], and as a bridge for hepatic transplantation [9, 10]. In this study, we were able to demonstrate a statistically significant difference between response rates according to RECIST versus mRECIST methods. The response rates were higher with mRECIST, which is consistent with results reported by Shim et al. in regard to tumor response assessments after TACE [16]. Moreover, mRECIST, like EASL, correlates better with survival than RECIST [16], and it is simpler to use than EASL, which needs bi-dimensional measurements. In keeping with previously published data [13, 17, 18], the local control rate in our study was very high, with only one infield progression. The outfield progression rate was 37%, which is somewhat lower than what was found in the study by Lo et al. [19]. This high rate can be explained by the cirrhotic disease, which may favor intra-hepatic occurrence of new lesions; as well as the local nature of SBRT treatment, like RFA.

Our study has highlighted the importance of post-treatment changes that have already been described on CT, but that have barely been studied by MRI. These include ring-like [20] T2-weighted high intensity and arterial enhanced signals, corresponding with an altered inflammatory zone with veno-occlusive damage [20]. These perilesional changes can mimic tumor progression, especially when the tumor arterial enhancement persists on the first imaging controls. However, there is no wash-out on the portal or delayed phases [21]. The post-SBRT changes are observable more than a year after treatment, albeit with less intensity and extension.

**Fig 7:**
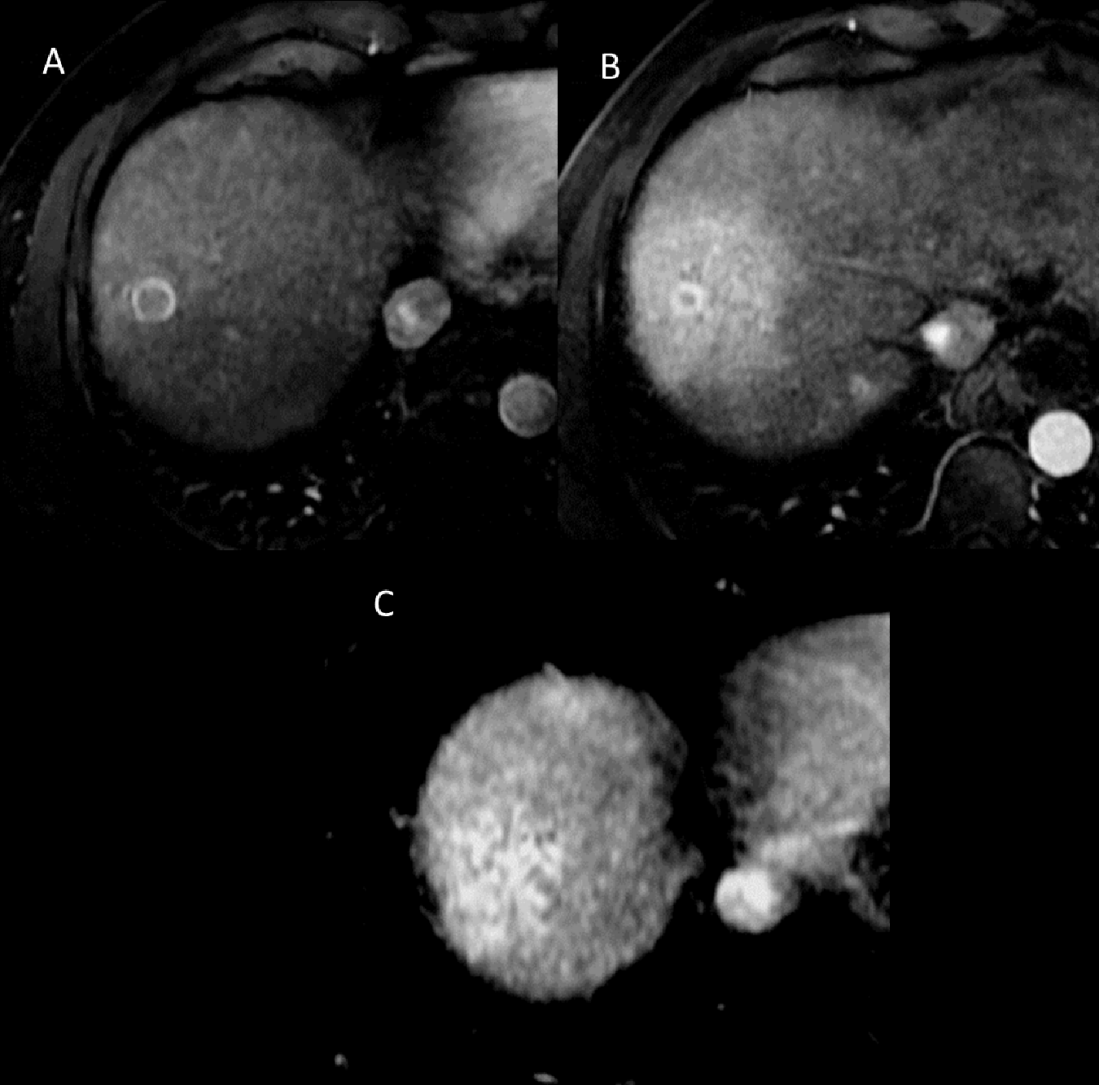
A 54-year-old man with HCC. 7A.Segment VIII HCC on arterial phase on dynamic 3D T1 weighted imaging at the time of diagnosis. 7B. Tumor regression at three months after treatment on arterial phase on dynamic 3D T1 weighted imaging. Target-like image with tumor annular enhancement and peritumoral ring-like enhanced post-treatment changes. 7C. Complete regression of the tumor at six months after SBRT on arterial phase on dynamic 3D T1 weighted imaging with persistent enhanced post-treatment changes.

The main aim of this study was to identify predictive factors for tumor responses after SBRT. Regression of the tumor arterial enhancement may indeed be variable and long-lasting [13] (Fig 7). We demonstrated that current T2-weighted and diffusion-weighted signals are a predictive factor for tumor responses in subsequent MRIs, according to RECIST. Thus, the absence of tumor diffusion-weighted hyperintensity on the MRI correlates with the probability of a complete response on the subsequent MRI three months later. The correlation was even better with the absence of tumor T2-weighted hyperintensity.

As for the predictive factors for tumor responses to SBRT at baseline, the size of the lesion and the diffusion-weighted signal were the only significant predictor. Contrary to what has been reported by Huang et al. [22], Child-Pugh scores did not exhibit potential as a prognostic factor, although it must be kept in mind that there were few patients with a score higher than A in our study. The size of the lesion has previously been shown to be a key element in the SBRT response, and lesions < 3 cm exhibited better results [22, 23]. Our study confirms these data, with a cut-off at 20 mm. These results are in favor of early treatment with SBRT.

T2-weighted intensity on pre-treatment MRI did not prove to be a predictive factor, although this may be due to the limited number of low intensity lesions in our cohort. However, the absence of diffusion-weighted hyperintensity on the pre-treatment images could be correlated with better responses according to RECIST and thus might be a deciding factor in choosing an ablative treatment.

Our study has several limitations. Firstly, there was a degree of variability in terms of the equipment that was used, since not all of the imaging was performed with the same MRI instrument. By using the same sequence protocol throughout, we were, however, able to ascertain the independence of the results in terms of the type of equipment or diffusion sequence that was used.

Secondly, this study lacks histological confirmation. Although the absence of radiological enhancement does not allow for a highly precise distinction to be made between viable lesions and necrosis [24], the use of radiological criteria is generally considered to have ample clinical value in regard to the assessment of treatment responses [16]. Lastly, the median follow-up was somewhat shorter than might be desired due to a patient who was lost from the study.

SBRT is a promising technique for the treatment of inoperable HCC. Post-treatment changes on MR imaging must, however, be interpreted with caution since they can resemble tumor progression.

The regression of tumor enhancement after treatment is variable over time. Diffusion-weighted and primarily T2-weighted intensities are predictive factors for treatment responses on subsequent MRIs, thus allowing predictions to be made in regard to tumor progression.

## Supporting information

S1 Table Data of the study. This table shows all data of patients and MRI.
(XLSX)
